# Comparison of two isolation methods of tobacco-derived extracellular vesicles, their characterization and uptake by plant and rat cells

**DOI:** 10.1038/s41598-022-23961-9

**Published:** 2022-11-18

**Authors:** Michaela Kocholata, Michaela Prusova, Hana Auer Malinska, Jan Maly, Olga Janouskova

**Affiliations:** 1 Centre for Nanomaterials and Biotechnology, Faculty of Science, Jan Evangelista Purkyne University in Usti nad Labem, Usti nad Labem, Czech Republic; 2 Department of Biology, Faculty of Science, Jan Evangelista Purkyne University in Usti nad Labem, Usti nad Labem, Czech Republic

**Keywords:** Cancer, Cell biology, Physiology, Plant sciences

## Abstract

Plant extracellular vesicles (pEVs) derived from numerous edible sources gain a lot of attention in recent years, mainly due to the potential to efficiently carry bioactive molecules into mammalian cells. In the present study, we focus on isolation of PDNVs (plant-derived nanovesicles) and pEVs from callus culture and from BY-2 culture of *Nicotiana tabacum* (tobacco). Tobacco was selected as a source of plant vesicles, as it is commonly used by human, moreover it is a model organism with established techniques for cultivation of explant cultures in vitro. Explant cultures are suitable for the isolation of pEVs in large quantities, due to their fast growth in sterile conditions. As the efficiency of isolation methods varies, we were comparing two methods of isolation. We evaluated biophysical and biochemical properties of plant vesicles, as well as differences between isolates. We encountered difficulties in the form of vesicles aggregation, which is often described in publications focused on mammalian nanovesicles. In an effort to prevent vesicle aggregation, we used trehalose in different stages of isolation. We show tobacco-derived vesicles successfully enter tobacco and mesenchymal cell lines. We observed that tobacco-nanovesicles isolated by different methods incorporated fluorescent dye with different efficiency. The results of our study show tobacco-derived vesicles isolated by various isolation methods are able to enter plant, as well as mammalian cells.

## Introduction

Extracellular vesicles (EVs) are small nanoscale membrane particles produced by various organisms. For a long time it has been believed that plants cannot produce extracellular vesicles due to the presence of cell wall. In recent years, this assumption was proven to be wrong and today it is generally accepted, that plants, as well as mammals, fungi, bacteria etc. can not only release EVs, but they can also internalize vesicles produced by other cells^1^. Plant extracellular vesicles are implicated in intercellular communication through transportation of bioactive cargo, including specific proteins, lipids, small RNAs and plant metabolites. Proteomic and metabolic analysis have revealed that plant vesicles can carry various proteins with antifungal and antimicrobial activity, as well as metabolites with antioxidant, anti-inflammatory or/and antitumor effects^2–8^. Additionally, pEVs participate in distance gene regulation and horizontal transfer of RNAs^9,10^. Due to their natural properties, plant nanovesicles can provide controlled and specific transport of bioactive molecules between cells^11–13^.

Plant EVs were proven to be involved in plant defense reactions and also in the interaction between plant and other organism, such as fungi, bacteria or human^6,11,14–16^. It has been presented that pEVs can internalize into mammalian (including human) cells, and thus can regulate a number of cellular processes. The resulting effects vary depending on the source of pEVs and on the vesicle cargo molecules^17^. While some studies have shown pEVs have antitumor effects, due to their immunomodulatory and antiproliferative properties, other studies have shown plant vesicles can serve as an efficient system for transportation of small molecules, including drugs^18,19^. Recent research of pEVs derived from *Zingiber officinale* (ginger), *Brassica oleracea var. italica* (broccoli), *Citrus aurantium* (grapefruit*), Lentinula edodes* (shiitake), *Vaccinium myrtillus* (blueberries) and *Vitis vinifera* (grapes) demonstrated that these vesicles participate in the reduction of inflammation and in immunomodulatory processes. The effects were observed mainly in vitro and on mice model suffering with inflammatory diseases, such as colitis^3,20–23^. Similarly, antitumor properties were observed in nanovesicles derived from *Citrus limon* (lemon), *Citrus aurantium* (bitter orange), *Panax ginseng*, *Dendropanax morbifera*, *Pinus densiflora* and *Moringa oleifera*^10,24–27^*.*

The structure of extracellular vesicles allows the protection and transportation of cargo molecules from one cell to another. Thus, not only naturally occurring plant-derived molecules, but also different types of drugs can be transported via pEVs^12,28,29^. In recent years, the interest in plant nanovesicles have raised due to the possibility of their use as a drug delivery system. Although human vesicles can be used for a drug transportation as well, there is a number of limitations associated with their use, such as a particular concern about how to generate a sufficient amount of human EVs in vitro or by isolation from biological fluids. Thus, plants can provide a good alternative, as it is possible to ensure continuous and fast growth of plant material, high yield of extracellular vesicles and it also provides non-toxic, stable and effective tool for transportation^30^. Although a number of studies have focused on plant extracellular vesicles and their effects on mammalian cells in vitro and in vivo, there were no toxic effects observed. Moreover, their presence didn’t trigger the immune response in mammalian cells^29,31^. Two types of pEVs nanovectors were used so far, with one being natural extracellular vesicles, and the other being pEVs-derived nanovectors, which are usually designed using the membrane of plant vesicles. Both can be loaded with specific drug or small RNAs and specifically transported into the place of interest, by tailoring their surface using chemical modification or genetic engineering^13^. Wang et al., coated grapefruit-derived nanovesicles with inflammatory related receptor-enriched membranes of activated leukocytes, as activated immune cells are able to target inflammatory sites with high efficiency. Modified vesicles were able effectively target inflammatory colon and breast cancer tissues^32^. Another group incorporated phosphatidic acid into lipid membranes of pEVs derived from ginger to achieve higher target specifity. Phosphatidic acid serves as a ligand of FR-receptors that are overexpressed in many tumors^33^. Li et al.^34^ demonstrated that ginger-derived vesicles functionalized with arrowtail pRNA-3WJ and folic acid for ligand display were able to deliver surviving siRNA to a KB cancer model, leading to an tumor growth inhibition.

Some pEVs have also been used to targeted delivery of antitumor drugs, including doxorubicin, paclitaxel or methotrexate into tumor sites, leading to an efficient and specific drug transportation, as well as reduction of systemic drug cytotoxicity^15,35–37^.

However the mechanism used by plant derived vesicles to enter cells has not yet been fully understood, several studies have observed the uptake of pEVs by mammalian cells, showing pEVs were taken up by clathrin-mediated endocytosis and macropinocytosis^20,36,38^.

To achieve the best results in trans-kingdom transmission, it is necessary to choose the optimal isolation method as well as plant material in a first place. Most frequently used method for isolation of pEVs is ultracentrifugation. The process begins with a series of purifying steps to remove cells and impurities. The last purification centrifugation step is followed by high-speed ultracentrifugation, usually at 40,000–200,000 × g, which sediments EVs^24,39–41^. Ultracentrifugation can be also supplemented by ultrafiltration, using filters with different pore size to capture contaminants of the sample^40,42,43^. In recent years, studies isolating pEVs using polyethylene glycol (PEG) precipitation have emerged. PEG solution allows EVs to precipitate and separate during low-speed centrifugation^44^. Obtained isolates usually contain high amount of contaminants, mainly proteins. Lately, studies adding “cleaning” steps into PEG-isolating protocol have emerged, promising the reduction of protein contaminants^44–46^.

Plant vesicles are mostly isolated from plant juice, apoplastic fluid, homogenized whole plants, but also plant explants cultures. The advantage of using explants cultures, such as callus or suspension cell cultures is the continuous and fast growth in sterile conditions^40,47^. Due to the lack of knowledge about pEVs biogenesis pathways, we deal with difficulties with their classification. As it was already pointed out by Pinedo et al. (2020), we can find in the literature number of different terms for plant vesicles, including microvesicles, nanovesicles, exosomes and exosome-like vesicles. They suggest the term “extracellular vesicles” for vesicles isolated exclusively from extracellular fluids. In contrary, when vesicles isolation method results in the rapture of cell or the tissue of the plant, leading to an increase of intracellular contamination, authors suggest the term “plant-derived nanovesicles” to be used to establish differences between these two groups of plant vesicles with different origin^17^. Therefore, we use the term “pEVs” only for vesicles isolated from tobacco suspension culture media and “PDNVs” for vesicles isolated from tobacco callus homogenate.

In this work, we isolated EVs and PDNVs from rarely used plant material, namely tobacco callus and tobacco suspension cells. We compared two methods of vesicles isolation—ultracentrifugation and polyethylene glycol precipitation. Size and vesicle concentration, as well as the presence of exosomal marker HSP70 were analyzed in isolated vesicles. Last but not least, the ability of tobacco vesicles to enter both plant and mammalian cells was observed.

## Materials and methods

### Plant material

BY-2 suspension culture of *Nicotiana tabacum* was provided by the Institute of Experimental Botany of the Czech Academy of Science. BY-2 cultures were cultivated in the dark at 26 °C on a shaker (105 RPM). Cells were passaged into fresh growth medium weekly. The medium was prepared using Murashige-Skoog (MS) basal salt (4.3 g/l), supplemented with KH_2_PO_4_ (0.2 g/l), saccharose (30 g/l), inositol (0.1 g/l), thiamine (1 mg/l) and 2,4-Dichlorophenoxyacetic acid (2,4-D; 0.2 mg/l). The pH of the medium was adjusted to 5.6–5.8 and the medium was sterilized in an autoclave at 121 °C for 20 min.

To induce callus formation, *Nicotiana tabacum* seeds, which were purchased at gardening store, were sterilized and placed on callus-inducing Murashige-Skoog medium supplemented with saccharose (30 g/l), plant agar (8 g/l), 1-Naphthaleneacetic acid (NAA; 1.2 mg/l) and 6-Benzylaminopurine (BAP; 0.12 μl/l).The pH of the medium was adjusted to 5.6–5.8 and the medium was sterilized in an autoclave at 121 °C for 20 min. Cultures were cultivated in phytotron under light at 25 °C. The formation of callus started in 3–4 weeks and callus was then transferred to fresh medium every 6 weeks.

### PEVs and PDNVs isolation

Tobacco-derived vesicles were isolated from callus and BY-2 suspension cultures using ultracentrifugation or polyethylene glycol precipitation. Tobacco vesicles were isolated from 15 ml of BY-2 suspension culture and from 15 ml of callus-homogenate obtained by the homogenization of 30 g of callus in 15 ml of PBS/PBS with trehalose using ultracentrifugation. For polyethylene glycol precipitation we used 0.5 ml of 10 000 × g supernatant from BY-2 suspension culture or callus-homogenate.

#### Ultracentrifugation

BY-2 suspension culture was collected 7 days after the transfer to fresh medium. The sample was centrifuged at 2000 × g for 20 min, at 4 °C, the supernatant was collected and centrifuged again at 10,000 × g for 30 min, at 4 °C, using Beckman Coulter´s Avanti JXN-26 high-speed centrifuge, rotor JA-25.50. Supernatant was than collected and centrifuged at 100,000 × g for 2 h, at 4 °C, using Beckman´s Coulter OPTIMA XPN 90 ultracentrifuge, rotor 70-Ti. The pellet was resuspended in 500 μl PBS/25 mM trehalose in PBS. Samples were stored at – 20 °C until further analysis.

Six weeks after the passage, 30 g of calluses were collected and homogenized in mortar with the addition of 15 ml of PBS/25 mM trehalose in PBS. The mixture was left at room temperature for 10 min. Subsequently, large particles were removed using sieve. The further isolation procedure was identical to BY-2-derived vesicles isolation steps. The pellet was resuspended in 500 μl PBS/25 mM trehalose in PBS. Samples were stored at − 20 °C.

Due to the problem with pellet resuspension, samples were filtered using 0.22 μm pore size filters before further analysis.

#### Trehalose addition

We tested the effects of trehalose on tobacco-vesicles aggregation, when added at different stages of vesicles isolation. Samples isolated by ultracentrifugation (a) did not contain trehalose or (b) trehalose was added at the beginning of isolation into BY-2 MS medium to trehalose final concentration of 25 mM. When callus was used as a source of vesicles, 25 mM trehalose-supplemented PBS was used for callus homogenization. Simultaneously, 100,000 × g ultracentrifugation pellet was resuspended in 25 mM trehalose in PBS. And finally, (c) only 100,000 × g centrifugation pellet was resuspended in 25 mM trehalose in PBS.

#### Polyethylene glycol precipitation

There was no trehalose added to the samples isolated by polyethylene glycol precipitation as the aggregation wasn’t significant.

BY-2 suspension culture was collected 7 days after the transfer to fresh medium. In case when trehalose was added to the sample at the beginning of the isolation, the required amount of trehalose was added directly to the culture where it was dissolved. The sample was centrifuged at 2000 × g for 20 min, at 4 °C, the supernatant was collected and centrifuged again at 10,000 × g for 30 min at 4 °C, using Beckman Coulter’s Avanti JXN-26 high-speed centrifuge, rotor JA-25.50. Supernatants were used for isolation using Aqueous Two Phase System (ATPS), described in Kirbas et al.^46^. Briefly, ATPS isolation solution was created by mixing polyethylene glycol and dextran into solution. Sample was added to the mixture in 1:1 ratio and centrifuged at 1000 × g, 10 min. Twice, upper phase was removed and replaced with the upper phase of washing solution (prepared by isolation solution mixed with dH_2_O in 1:1 ratio). Samples were centrifuged again at 1000 × g for 10 min. After the last washing step, the upper phase was removed and the PEG-rich phase containing tobacco-derived vesicles was obtained. Samples were stored at − 20 °C.

Six week after the passage, 30 g of calluses were collected and crushed in mortar with the addition of 15 ml of PBS or 15 ml of 25 mM trehalose in PBS. The mixture was left at room temperature for 10 min. The further isolation procedure was identical to BY-2-derived vesicles ATPS isolation steps described above. Isolated vesicles were stored at − 20 °C.

### Dynamic light scattering

Dynamic Light Scattering (DLS) analyses were performed using Malvern Instruments’ Zetasizer Nano and cuvettes (ZEN0040). Approximately, 300 μl of the sample was used for analysis. Sample was calibrated at 25 °C for 120 s, followed by three readings consisting of 8 measurements each. The ambient temperature did not exceed 25 °C.

### Nanoparticle tracking analysis

Particles present in the sample were analyzed using Malvern Instruments’ NanoSight NS 3000 and videos were collected and analyzed by the NTA software. Approximately, 300–400 μl of each sample was loaded into flow-cell top-plate chamber. A laser beam with λ = 562 nm illuminated the chamber from the bottom and the light was scattered by the particles present in the solution. Each sample was analyzed three times for 60 s; the ambient temperature did not exceed 25 °C. Results were assessed using the Malvern software.

### Protein concentration assay

A BCA (bicinchoninic acid) Protein Assay Kit (Merck) was used for a protein concentration assay. A standard curve (0–2000 μg/ml) was derived with six points of serial dilution of bovine serum albumin (BSA) and a working reagent. All samples were replicated three times. 25 μl of each sample was mixed with 200 μl of working reagent and incubated for 30 min at 37 °C. Another sets of measurements were performed with 20 μl of each sample with addition of 5 μl of RIPA 5×, 200 μl of working reagent was added and samples were incubated for 30 min at 37 °C. After cooling to room temperature, the absorbance at 562 nm was measured using GloMax Discover Microplate Reader and a protein concentration was calculated using the standard curve.

### Rat mesenchymal stem cells

The rat mesenchymal stem cells (rMSCs) were kindly provided by Dr. P. Jendelova, Institute of Experimental Medicine of the Czech Academy of Sciences, and were cultivated in DMEM supplemented with fetal bovine serum (FBS), 100 units of penicillin, and 100 µg/ml streptomycin in 25 cm^2^ flask.

### Confocal microscopy and labeling of vesicles

To detect the incorporation of tobacco-derived vesicles into various cells, vesicles were labeled using Bodipy TR Ceramide and incubated with *Nicotiana tabacum* cells and rat mesenchymal stem cells. Fluorescent signal was observed using confocal microscopy.

Bodipy TR Ceramide is a red-fluorescent dye that can be used to stain Golgi apparatus in live cells as well as natural biologically active sphingolipids.

Per 100 μl of pEVs/PDNVs sample (1 × 10^8^ particles/sample) 1 μl of 1 mM Bodipy TR Ceramide was added, followed by the incubation at 37 °C, 25 min. The excess dye was washed out using 100,000 × g (1 h) ultracentrifugation or using Exosome Spin Columns (Thermo Fisher Scientific). Stained pEVs/PDNVs were incubated with tobacco cells (1 × 10^5^) in culture medium for 4 h at the shaker (105 RPM), 26 °C. Subsequently, cells were purified by replacing the medium twice, using centrifugation. Then 1 ml of cells in culture medium was added to the glass bottom 35 mm dish with 20 mm bottom well (Thermo Fisher Scientific) and observed using confocal laser scanning microscope (Leica SP8).

The same procedure was repeated with rat mesenchymal stem cells. The amount of 1 × 10^5^ of rat mesenchymal stem cells in 1 ml of DMEM supplemented with 10% FBS and P/S was plated into the glass bottom 35 mm dish with 20 mm bottom well (Thermo Scientific). Following day, per 100 μl of tobacco-derived vecisles sample (1 × 10^8^ particles/sample) 1 μl of 1 mM Bodipy TR Ceramide was added, followed by the incubation at 37 °C for 25 min. The excess dye was washed out using 100,000 × g (1 h) ultracentrifugation or using Exosome Spin Columns (Thermo Fisher Scientific). Stained plant vesicles were incubated with rat mesenchymal stem cells on the glass bottom dish at 37 °C for 4 h, than the cells were purified by replacing the medium twice. Purified cells were observed using confocal laser scanning microscope.

### Spectrofluorimetry

We used spectrofluorimetric analysis to verify whether the dye (Bodipy TR Ceramide) is incorporated into various tobacco-derived vesicles with different efficiency. We isolated three samples from callus and BY-2 cell culture using ultracentrifugation as well as polyethylene glycol precipitation. We incubated the same concentration of vesicles from each sample with Bodipy TR Ceramide for 25 min at 37 °C, followed by ultracentrifugation at 100,000 × g for 1 h at 4 °C, using Beckman Coulter’s Avanti JXN-26 high-speed centrifuge, rotor JA-25.50 as a cleaning step. Pellets were resuspended in 100 µl of PBS. The fluorescence intensity was analyzed using Spectrofluorimeter (Horiba, FluoroMax 4).

### Western blotting

We performed western blotting using three different lysis buffers (a–c). The (a) buffer was composed of RIPA 1× and PMSF (5 mM); the composition of buffer (b) was the same, but with addition of β-mercaptoethanol (5%). Buffer (c) was composed of urea (21 M), phenylmethylsulfonyl fluoride (PMSF; 5 mM) and dithiothreitol (DTT; 1%). EVs lysates were subjected to SDS-PAGE on 12% polyacrylamide gel. Proteins were blotted onto nitrocellulose membrane 0.45 μm (Bio-Rad). After blocking in EveryBlot Blocking Buffer (Bio-Rad, 12010020) the membrane was washed three times in TBST 1× and incubated with rabbit Anti-HSP70 (Agrisera) antibody (1:1000) overnight at 4 °C. The membrane was further washed three times and incubated with horseradish peroxidase-conjugated goat Anti-Rabbit IgG (Agrisera; H+L; 1:2000) at room temperature for 1 h. Bands were detected using Novex chromogenic substrate (Thermo Fisher Scientific) according to the instructions.

### Statistical analysis

Statistical analysis was performed using Prism GraphPad Software. T-test was used to analyze the significance of differences between various samples. Statistical significance was accepted if p < 0.05.

All experiments were performed in accordance with relevant guidelines and regulations.

## Results and discussion

### Characterization of *N. tabacum*-derived vesicles

In the present study, we isolated plant extracellular vesicles from BY-2 suspension cultures and plant-derived nanovesicles from tobacco callus cultures. pEVs were isolated directly from the culture media in case of BY-2 suspension culture, and PDNVs were isolated from callus cells. The advantage of this type of plant material is its fast and continuous growth, which makes it a constant source of vesicles in large quantities. Moreover, the yields of plant vesicles obtained by using plant explant cultures are high. Whereas there is no standardized protocol, we decided to use and compare two methods for vesicle isolation—ultracentrifugation and polyethylene glycol precipitation. Initially, isolated vesicles were characterized using DLS. Since our samples were highly polydisperse, the Z-Average value significantly changed in time and it also differed from the size of individual fractions, we suggested tobacco-vesicles isolated by ultracentrifugation from both types of plant material could form aggregates. Our assumption was supported by the literature, where the aggregation of exosomes is mentioned, especially for exosomes of mammalian origin^48,49^. Due to the limitation of DLS measurements, as a second method to evaluate pEVs and PDNVs size and concentration NTA was used. Using NTA, we were able to characterize isolated vesicles and also visually confirm their aggregation, as shown in supplementary Fig. S1. In an effort to eliminate the presence of tobacco-derived vesicles aggregates, we examined the effects of trehalose (see Tables 1 and 2). We investigated a difference in vesicles size and concentration, protein concentration and aggregation in dependence of isolation method (ultracentrifugation vs. polyethylene glycol precipitation). Moreover we evaluated the influence of trehalose on the aggregation of samples with no trehalose (X), trehalose added at the beginning of the isolation (B) and for pelet resuspention (P), when isolated using ultracentrifugation. Trehalose is used to eliminate the presence of plant-derived vesicles aggregates in mammalian and plant exosomes^48,49^.

**Table 1:** The characterization of pEVs and PDNVs isolated by ultracentrifugation from BY-2 and callus cultures of tobacco.

Ultracentrifugation		DLS	NTA	NTA	BCA
Sample origine	Trehalose	Z-AverageDiameter (nm)	Size (nm)	Concentracion (particles/ml)	Protein concentration (μg/ml)
Callus	X	631.4 ± 158.0 (□)	209.3 ± 10.85	1.71 × 10^9^ ± 3.46 × 10^8^	96.19–506.18
	P	380.4 ± 136.70 (*)	214.7 ± 9.26	3.61 × 10^9^ ± 1.50 × 10^9^	98.14–482.86
	B	967.6 ± 63.14 (*, ○)	253.2 ± 25.31	1.86 × 10^9^ ± 1.61 × 10^9^	179.04–486.50
BY-2 suspension culture	X	565.1 ± 220.7	175.5 ± 14.45	4.69 × 10^9^ ± 1.06 × 10^9^	713.60–1147.06
	P	354.2 ± 66.85 (∆)	301.9 ± 115.90	7.98 × 10^9^ ± 4.52 × 10^9^	732.28–1046.90
	B	681.0 ± 143.30 (⌂)	352.8 ± 92.57	2.18 × 10^9^ ± 1.80 × 10^9^	552.75–983.61

The comparison of the vesicles sizes using Dynamic Light Scattering (DLS) and Nanoparticle Tracking analysis (NTA) and protein concentration. X—no trehalose added, P—100,000 × g ultracentrifugation pellet resuspended in 25 mM trehalose in PBS, B—25 mM trehalose added at the beginning of the isolation and also for the resuspension of 100,000 × g ultracentrifugation pellet. Marks (□,*, ○, ∆, ⌂) written behind the Z-Average values indicate samples which Z-Average size differences are significant.

**Table 2:** The characterization of pEVs and PDNVs isolated by polyethylene glycol precipitation from BY-2 and callus cultures of tobacco.

PEG precipitation		DLS	NTA	NTA	BCA
Sample origin	Trehalose	Z-average diameter (nm)	Size (nm)	Concentration (particles/ml)	Protein concentration (μg/ml)
Callus	X	116.70 ± 8.67 (□, ○)	209.9 ± 0.38	0.55 × 10^9^ ± 1.75 × 10^8^	1843.69 – 2073.56
BY-2 suspension culture	X	138.70 ± 32.80 (∆, ⌂)	205.6 ± 10.84	2.63 × 10^9^ ± 1.25 × 10^9^	106.24 – 318.78

The comparison of the vesicle size using Dynamic Light Scattering (DLS) and Nanoparticle Tracking analysis (NTA) and protein concentration. X—no trehalose added.

#### Concentration, size and protein concentration of tobacco-derived vesicles

The physical characterization, including particle size and concentration was performed using DLS and NTA. As shown in Table 1, sizes obtained using DLS were larger than sizes obtained using NTA. NTA, as well as DLS, measures Brownian motion related to hydrodynamic diameter of the particle. NTA measures Brownian motion by image analysis, tracking the motion of each particle which is related to particle size. The method is more suitable for polydisperse samples as it analyzes each particle separately. In comparison, DLS doesn’t visualize particles, but it monitors fluctuations of the scatter intensity. Thus, the method “favors” large particles scattering the light with higher intensity which can lead to inaccurate average values. This is clearly evident on the differences between the Z-Averages and size values of our highly polydisperse samples, which were obtained using NTA (Table 1)^50^.

Different results were obtained for EVs isolated using polyethylene glycol precipitation. DLS Z-Average sizes were smaller, than sizes obtained by NTA analysis. Since the Z-Average value is given not only by the “core” particle, but also by ions bound to its surface, it is likely that the lower values of Z-Average in case of PEG-isolated vesicles may be due to the surface attached molecules of PEG, which “covers” the surface of tobacco-derived vesicles, possibly eliminating the presence of surface ions^51^.

The Z-Average values of the diameter of vesicles isolated by ultracentrifugation were 631.4 ± 158.0 nm; 380.4 ± 136.70 nm and 967.6 ± 63.14 nm for callus-derived vesicles X, P, B and 565.1 ± 220 nm; 354.2 ± 66.85 nm and 681.0 ± 143.30 nm for vesicles isolated from BY2 cultures X, P, B (Table 1). By PEG-isolation the Z-Average values reached sizes of 116.7 ± 8.674 nm for callus-derived PDNVs and 138.7 ± 32.80 nm for BY-2-derived vesicles (Table 2). Significant differences between individual sizes are shown in Tables 1 and 2 using various symbols. Although DLS has some limitations for analyzing certain types of samples, in our case it helped us to detect the presence of vesicles aggregates, as well as the effect of trehalose on vesicle aggregation. However we don’t recommend to use only DLS for EVs and PDNVs size analysis, as it can lead to misinterpretation of actual sizes of vesicles^7^. Our results show the presence of aggregates in samples with no trehalose (X), as the Z-Average diameter size is too high for exosomes and the average size has changed when trehalose was added to the solution. When trehalose was used for pellet resuspension (P), the average size slightly decreased. However, when trehalose was used since the beginning of the isolation (B), the average size was higher than in previous cases, indicating the presence of bigger aggregates. We suggest it may be due to the trehalose saturation, when added at the beginning of the isolation into the solution containing disrupted cells and other contaminants, leading to the reduction of its effect.

In comparison, there are no significant differences between sizes obtained by NTA, since the method uses a different size measurement principle, as mentioned above. The average size values for callus-derived vesicles X, P, B were 209.3 ± 10.85 nm; 214.7 ± 9.26 and 253.2 ± 25.31 nm. Vesicles isolated from BY2 cells reached sizes of 175.5 ± 14.45 nm; 301.9 ± 115.9 and 352.8 ± 92.57 nm (Table 1). Similarly, vesicles isolated by PEG precipitation reached sizes of 209.9 ± 0.38 nm for callus-derived vesicles and 205.6 ± 10.84 nm for vesicles isolated from BY-2 culture (Table 2). Although NTA provides more accurate data in case of polydisperse samples, the effect of trehalose is not as well visible as it is using DLS.

The highest concentration of nanovesicles was obtained from BY-2 suspension culture, using both, ultracentrifugation and polyethylene glycol precipitation. The concentration of vesicles in suspension was determined by NanoSight as number of particles per 1 ml of final sample containing tobacco-derived vesicles. Particle concentration reached 3.61 × 10^9^ particles/ml for callus (P), 1.71 × 10^9^ particles/ml for callus (X), 1.86 × 10^9^ particles/ml for callus (B) and 7.98 × 10^9^ particles/ml for BY-2 (P), 4.69 × 10^9^ particles/ml for BY-2 (X) and 2.18 × 10^9^ particles/ml for BY-2 (B). The concentration of vesicles isolated by PEG precipitation reached 5.49 × 10^8^ particles/ml for vesicles isolated from BY-2 culture and 2.63 × 10^9^ particles/ml for vesicles isolated from callus (Tables 1 and 2).

Our results show, that we were able to isolate plant extracellular vesicles and plant-derived nanovesicles using ultracentrifugation and polyethylene glycol precipitation from suspension and callus cultures of *N. tabacum*. It is evident, plant vesicles isolated using PEG precipitation do not tend to aggregate, compared to pEVs and PDNVs isolated by ultracentrifugation. Using the same isolation method, no aggregation was observed by other research groups^45,46^. The sizes measured by NTA and concentrations of isolated vesicles were similar for both methods of isolation. We observed that the addition of trehalose into samples didn’t fully resolve aggregation as we observed aggregates during NTA analysis in all samples, including samples with trehalose. Bosch et al.^49^ performed NTA on samples isolated using PBS or using PBS with trehalose (25 mM). Their results showed that the size median was similar (113 and 111 nm) in samples without and with trehalose, respectively. Also the concentration in samples significantly increased^49^. We obtained surprising results showing that measured particle sizes were the largest in case of samples, where the trehalose was added at the beginning, although we expected the opposite. Even though there is no significant difference between the sizes of the individual samples, the size difference is still larger than in the publication mentioned above. We hypothesize this may be due to the interaction of trehalose with various proteins at the beginning of the isolation, leading to the trehalose saturation. Also, co-isolation of trehalose-protein complexes could lead to the interaction between proteins and isolated vesicles, explaining the presence of aggregates in samples even after 0.22 μm filters were used.

#### Protein concentration and exosomal marker detection

The protein concentration assay was done using BCA Protein Assay Kit. We observed differences between samples isolated by different methods, and also between samples isolated from different plant material (see Tables 1 and 2). Since callus is formed of dedifferentiated cells, it is clear that the protein concentration of the cell and also callus-derived PDNVs may differ from protein concentration of differentiated BY-2-derived vesicles, which was supported by our findings. The difference between the lower and higher protein concentrations is fivefold in case of PDNVs from callus, but only doubled when isolated from BY-2 (Table 1). When vesicles were isolated using PEG precipitations, we observed higher differences between callus and BY-2 derived vesicles. It can be caused by the co-isolation of various proteins along with tobacco vesicles (Table 2).

Moreover, the principle of isolation method may support the co-isolation of various protein contaminants. When vesicles were isolated using PEG precipitation, usually resulting in protein contamination, there was a higher chance in protein contamination than when using ultracentrifugation. We observed higher protein concentrations of PEG-isolated callus-derived vesicles, which confirms that PDNVs isolation from complex material, such as plant callus, may lead to higher protein co-isolation when using this method than when isolated from suspense culture media. Since we isolated PDNVs from exact callus weight and pEVs from exact BY-2 suspension volume, we summarize the values of tobacco-derived vesicles protein concentrations in the range given by measuring the concentration in triplicates for each sample type (Tables 1 and 2). We presented a range of protein concentration values, as the plant material, even when weighed, may be very variable in number of cells, which can have different sizes as well as different vesicles production.

Vesicles isolated by ultracentrifugation from callus reached lower protein concentration, ranging from 96.19 to 506.18 μg/ml for samples with no trehalose, 98.14–482.86 μg/ml with trehalose added for pellet resuspension and 179.04–486.50 μg/ml when trehalose was added at the beginning of isolation. For vesicles isolated from suspension cultures, the protein concentration was as following: 713.60–1147.06 μg/ml with no trehalose, 732.28–1046.90 μg/ml when trehalose was added for pellet resuspension, and finally, 552.75–983.61 μg/ml when trehalose was added at the beginning. Different results were obtained when pEVs and PDNVs were isolated using polyethylene glycol precipitation. Callus-derived vesicles had higher protein concentration ranging from 1843.69 to 2073.56 μg/ml. On the other hand, protein concentration of vesicles isolated from suspension cultures was 106.24 to 318.78 μg/ml.

**Figure 1:**
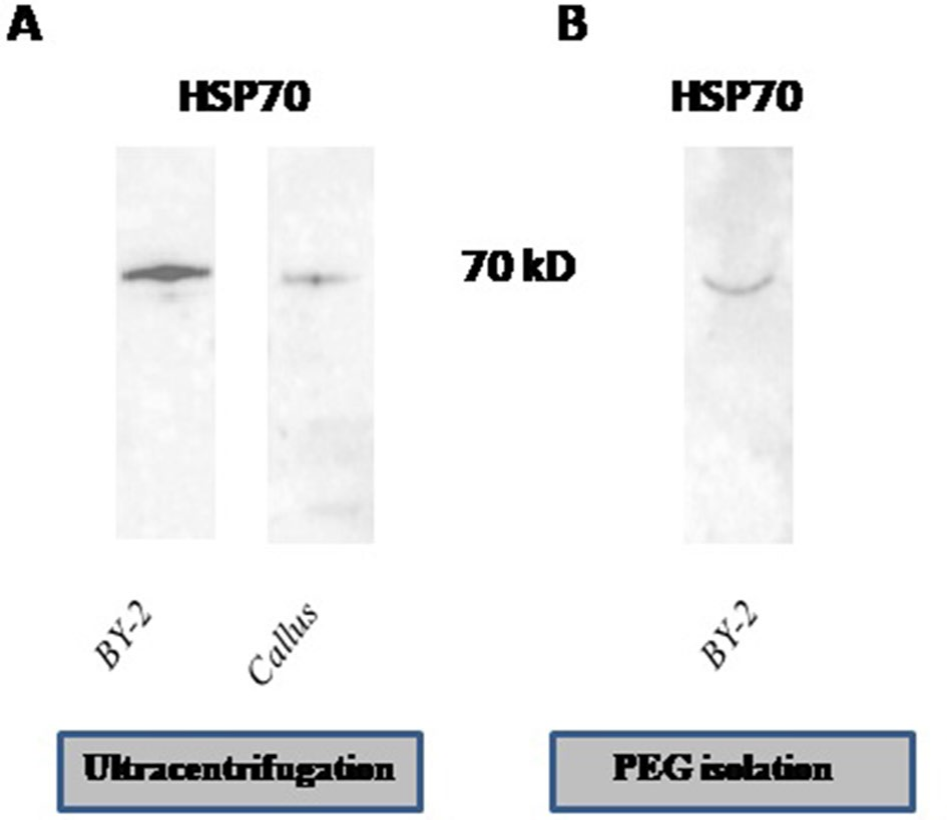
Detection of HSP70 in tobacco-derived vesicles. (**A**) Western blot analysis of HSP70 in vesicles isolated by ultracentrifugation, derived from BY-2 cultures and callus cultures. (**B**) Western blot analysis of HSP70 in vesicles isolated by polyethylene glycol precipitation, derived from BY-2 cultures. The lysis buffer was composed of RIPA 1X, PMSF (5 mM) and β-mercaptoethanol (5%). The results originate from one gel, the membrane is shown in Supplementary Fig. S3.

Due to the fact that the protein characterization of plant extracellular vesicles is not extensive, we estimated one potential marker in the literature that we were able to detect^17^. As there is no standardized protocol for pEVs marker detection, we compared the efficiency of three lysis buffers, as described in Methods. We investigated the presence of exosomal marker HSP70 (Fig. 1) in callus PDNVs and BY-2 cultures pEVs isolated using ultracentrifugation with no trehalose added, using three lysis buffers composed of: (a) RIPA 1X and PMSF (5 mM); (b) RIPA 1X and PMSF (5 mM), β-mercaptoethanol (5%); and (c) urea (21 M), phenylmethylsulfonyl fluoride (PMSF; 5 mM) and dithiothreitol (DTT; 1%). Based on our results, we selected buffer (b), as the background was the lowest (Supplementary Fig. S2), for further analysis of the presence of HSP70 in BY-2-derived pEVs and callus-derived PDNVs. Western Blotting confirmed the presence of HSP70 in vesicles isolated from *Nicotiana tabacum*. The presence of HSP70 was confirmed in callus-derived vesicles, as well as BY-2 suspension culture-derived pEVs.

#### The uptake of N. tabacum—derived vesicles by various cells

**Figure 2:**
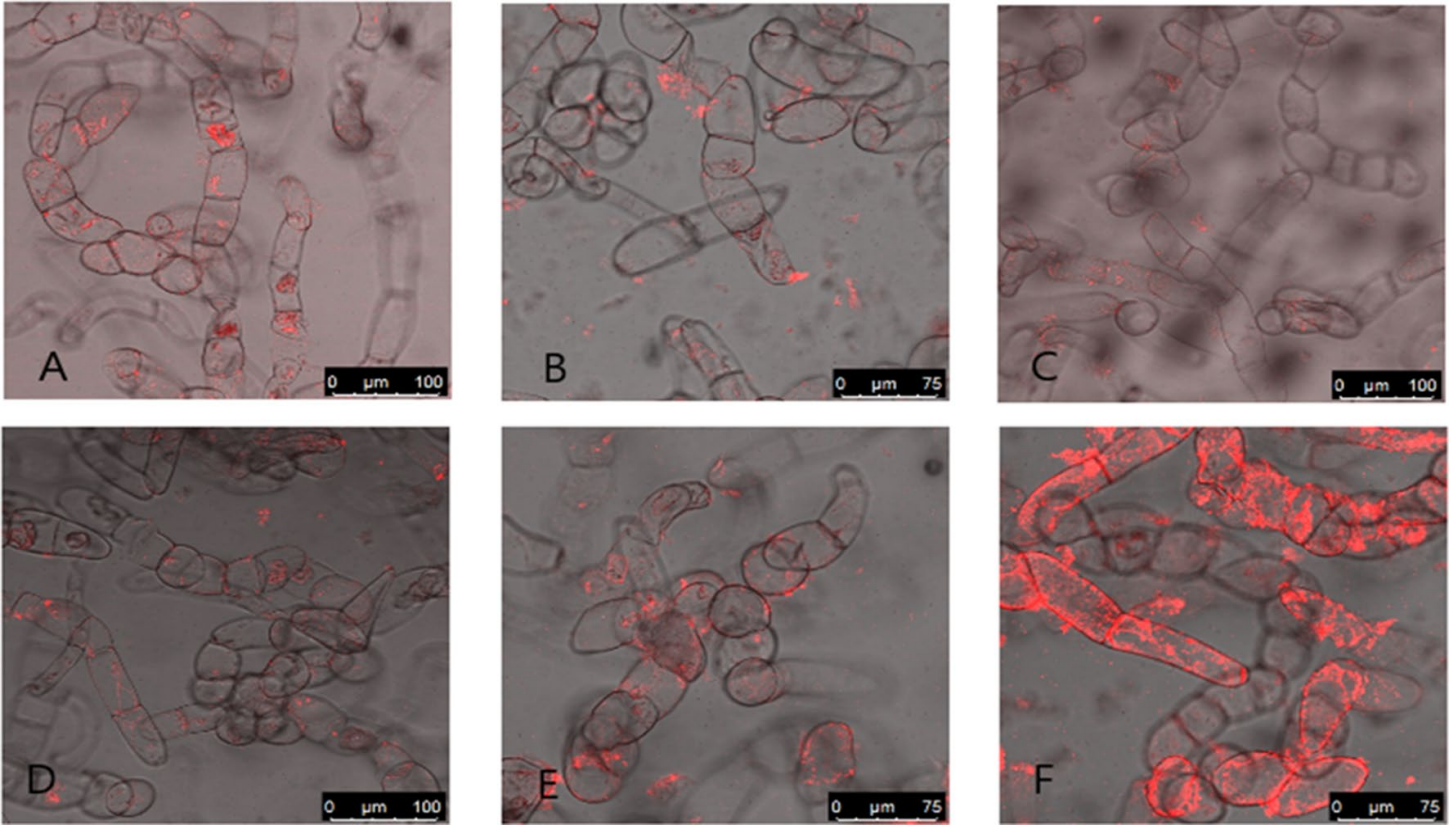
The uptake of tobacco vesicles by tobacco cells. (**A**) Vesicles isolated from callus with no trehalose added, (**B**) vesicles isolated from callus, pellet resuspended in PBS with trehalose, (**C**) samples isolated from BY-2, no trehalose added, (**D**) sample isolated from BY-2, trehalose was added for pellet resuspension. Samples (**E**) (callus) and (**F**) (BY-2) were isolated using PEG precipitation.

**Figure 3:**
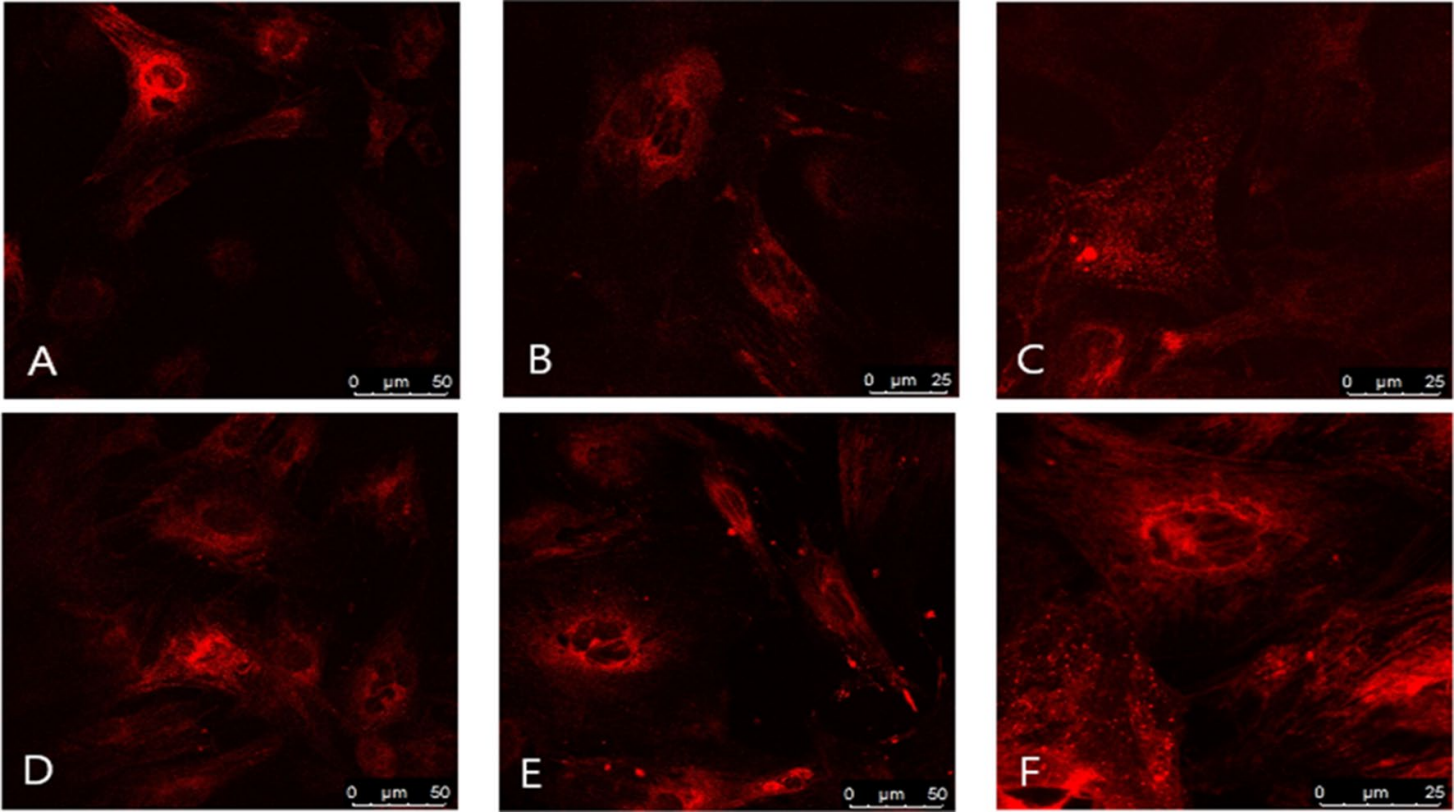
The uptake of tobacco vesicles by rat mesenchymal stem cells. (**A**) vesicles isolated from callus with no trehalose added, (**B**) vesicles isolated from callus, pellet resuspended in PBS with trehalose, (**C**) samples isolated from BY-2, no trehalose added, (**D**) sample isolated from BY-2, trehalose was added for pellet resuspension. Samples (**E**) (callus) and (**F**) (BY-2) were isolated using PEG precipitation.

**Figure 4:**
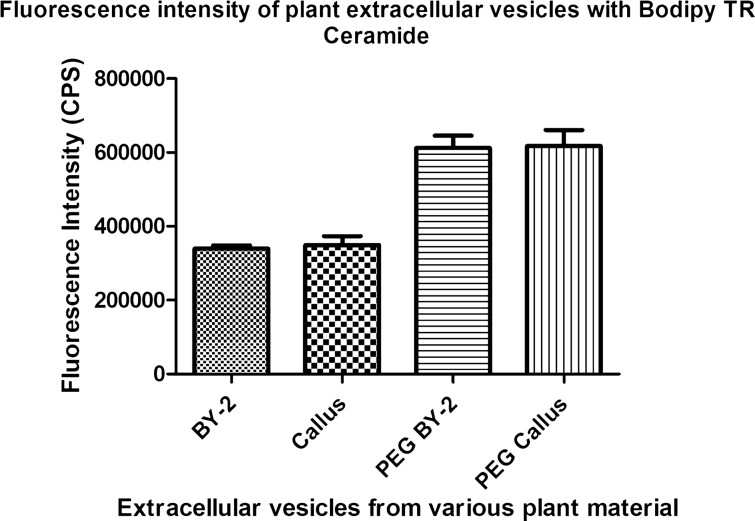
The fluorescence intensity of labeled vesicles. The figure shows fluorescence intensity of vesicles isolated by ultracentrifugation from BY-2 suspension culture (BY-2) and from callus (Callus), as well as vesicles isolated by polyethylene glycol precipitation from BY-2 cultures (PEG BY-2) and callus (PEG Callus).

To determine whether tobacco vesicles are able to enter cells, we labeled them using Bodipy TR Ceramide and incubated them with tobacco cells (BY-2) and rat mesenchymal stem cells (rMSCs). We performed various cleaning steps to remove unbound dye, including ultracentrifugation at 100,000 × g, for 1 h and using Exosome Spin Columns. We observed the uptake of tobacco-derived vesicles using confocal microscopy. Our observation showed higher fluorescence signals in some cell samples (Figs. 2 and 3). To evaluate whether it is due to different uptake ability or by different dye incorporation, we performed spectrofluorimetric analysis (Fig. 4).

The analysis of fluorescence intensity suggests, there is a significant difference in dye incorporation between samples isolated using ultracentrifugation and polyethylene glycol precipitation, as shown in Fig. 4. Our findings confirm the differences in the uptake efficiency, depending on the isolation method, affecting the signal intensity in the uptake of tobacco-derived vesicles by cells. Our study demonstrates that tobacco-derived vesicles are able to enter tobacco cells, as well as rat mesenchymal stem cells. Although the polyethylene glycol precipitation allows more efficient pEVs and PDNVs labeling, vesicles enter both cells types, but with different efficiency. This is not apparent in pEVs and PDNVs isolated by ultracentrifugation.

## Conculsions

In the present study tobacco calluses and suspension (BY-2) cells were used as a source of PDNVs and pEVs. This in vitro methodology can offer reproducible source of plant-derived vesicles for further applications, e.g. drug delivery. Moreover, two isolation methods were used and compared together with the application of trehalose for minimizing the effect of vesicles aggregation after their isolation using ultracentrifugation. Although trehalose decrease pEVs and PDNVs aggregation, its effect is dependent on the step of vesicles isolation when it is added. Our investigation show that adding trehlose during pellet resuspension lead to a sligh decrease of the average size of tobacco-derived vesicles. However, when trehalose was used since the beginning of the isolation, the average size was higher than in previous case, indicating the presence of bigger aggregates. We suggest it may be due to the trehalose saturation, when added at the beginning of the isolation into the solution containing disrupted cells and other contaminants, leading to the reduction of its effect. We have not been able to solve the aggregation of tobacco-derived vesicles using trehalose during their isolation by ultracentrifugation with complete efficiency. As we show in this study, pEVs and PDNVs tend to aggregate when isolated using ultracentrifugation, in contrast with polyethylene glycol precipitation isolates, where we did not observe vesicle aggregation. Although the method using polyethylene glycol is faster and produces vesicles of similar sizes and yields as ultracentrifugation, it appears that it may contribute to the co-isolation of contaminants, such as proteins.

Isolated vesicles were analyzed using DLS and NTA, showing differences between both isolation methods. Western blotting was used for the first time for proteins associated with extracellular vesicles of tobacco. We managed to detect exosomal protein marker HSP70 in vesicles isolated from tobacco. Finally, using plant cells and also rat mesenchymal cells we were able to confirm pEVs and PDNVs uptake into these cells. We detected differences in dye incorporation depending on the isolation method of tobacco vesicles. We observed higher incorporation of fluorescent dye into tobacco vesicles isolated by polyethylene glycol precipitation than into vesicles isolated by ultracentrifugation.

## Supplementary Information

Supplementary Figures.

## Data Availability

The datasets used and/or analysed during the current study available from the corresponding author on reasonable request.

## Abbreviations

ATPS: Aqueous Two Phase System
BAP: 6-Benzylaminopurine
BCA: Bicinchoninic acid
BSA: Bovineserum albumin
DLS: Dynamic Light Scattering
DTT: Dithiothreitol
EVs: Extracellular vesicles
HSP70: Heat Shock Protein 70
MS: Murashige-Skoog medium
NAA: 1-Naphthaleneacetic acid
NTA: Nanoparticle Tracking Analysis
PEG: Polyethylene glycol
PDNVs: Plant-derived nanovesicles
pEVs: Plant extracellular vesicles
PMSF: Phenylmethylsulfonyl fluoride
2,4-D: 2,4-Dichlorophenoxyacetic acid
