# Supplementary material for: Comparison of two isolation methods of tobacco-derived extracellular vesicles, their characterization and uptake by plant and rat cells
Source: Sci Rep. 2022 Nov 18;12:19896. doi: 10.1038/s41598-022-23961-9 (PMC9674704; doi:10.1038/s41598-022-23961-9)
Supplement: Supplementary file 1 — Supplementary Figures. [file 41598_2022_23961_MOESM1_ESM.pdf]

Comparison of two isolation methods of tobacco-derived extracellular vesicles, their characterization and uptake by plant and rat cells

Michaela Kocholata[1,*], Michaela Prusova[1], Hana Auer Malinska[2], Jan Maly[1] and Olga Janouskova[1]

[1]Centre for Nanomaterials and Biotechnology, Faculty of Science, Jan Evangelista Purkyne University in Usti nad Labem, Usti nad Labem, Czech Republic

[2]Department of Biology, Faculty of Science, Jan Evangelista Purkyne University in Usti nad Labem, Usti nad Labem, Czech Republic

*Correspondence: kocholata.michaela@gmail.com

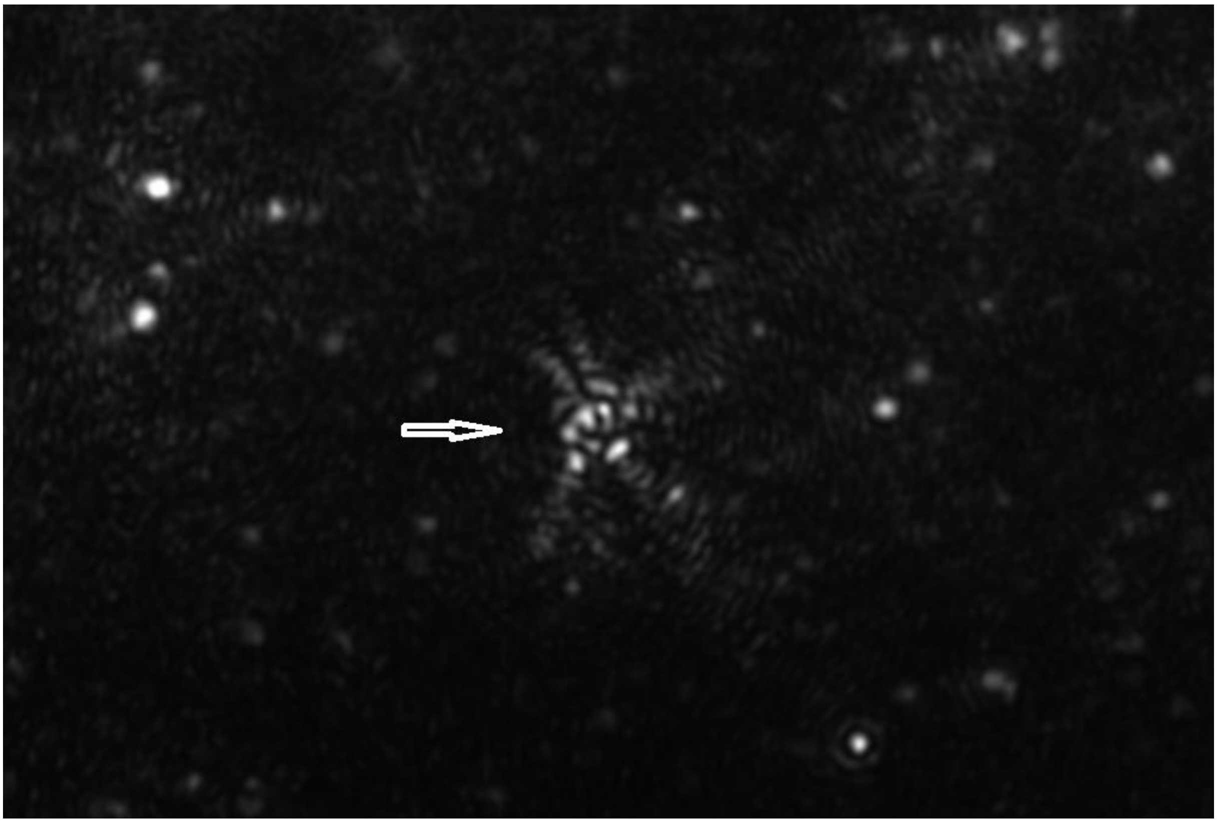

Supplementary Fig. S1: *A crop of a video captured by NanoSight NS300 (Malvern Instruments) showing the presence of extracellular vesicles agregate (marked with an arrow).*

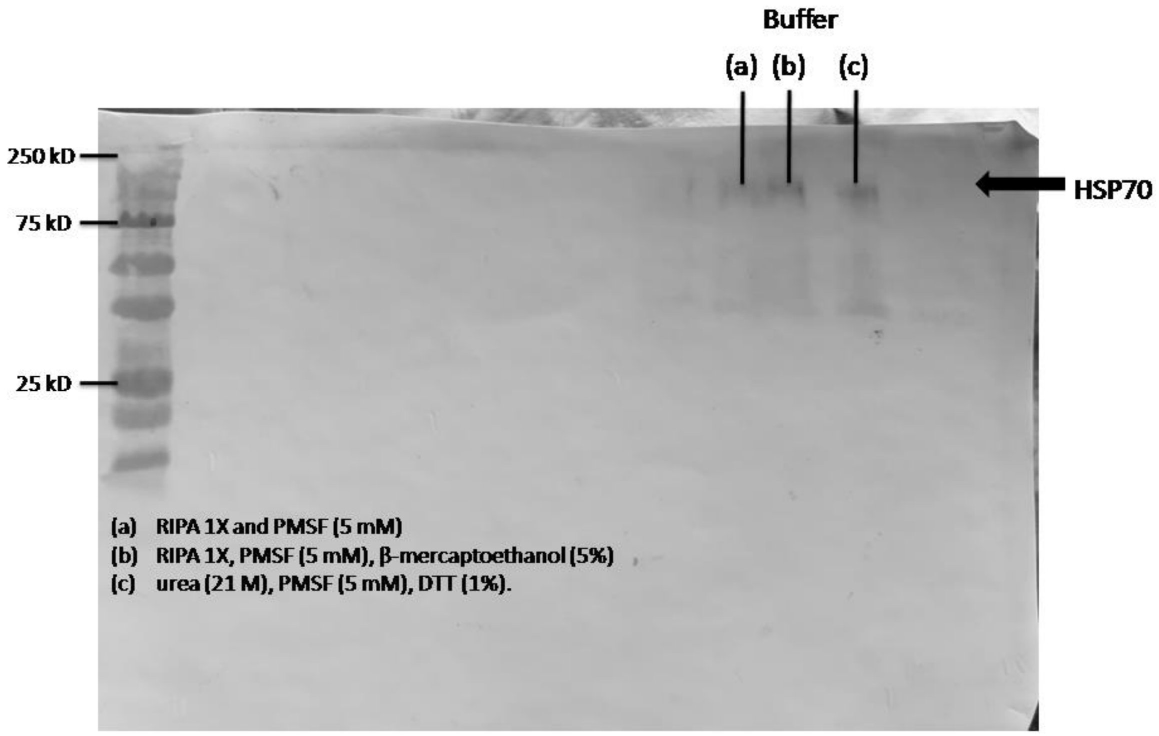

Supplementary Fig. S2: *Nitrocellulose membrane claiming the presence of exosomal protein marker HSP70 in extracellular vesicles isolated from tobacco BY-2 suspension culture, using three different lysis buffers. Buffer (b) was selected for further western blot analysis.*

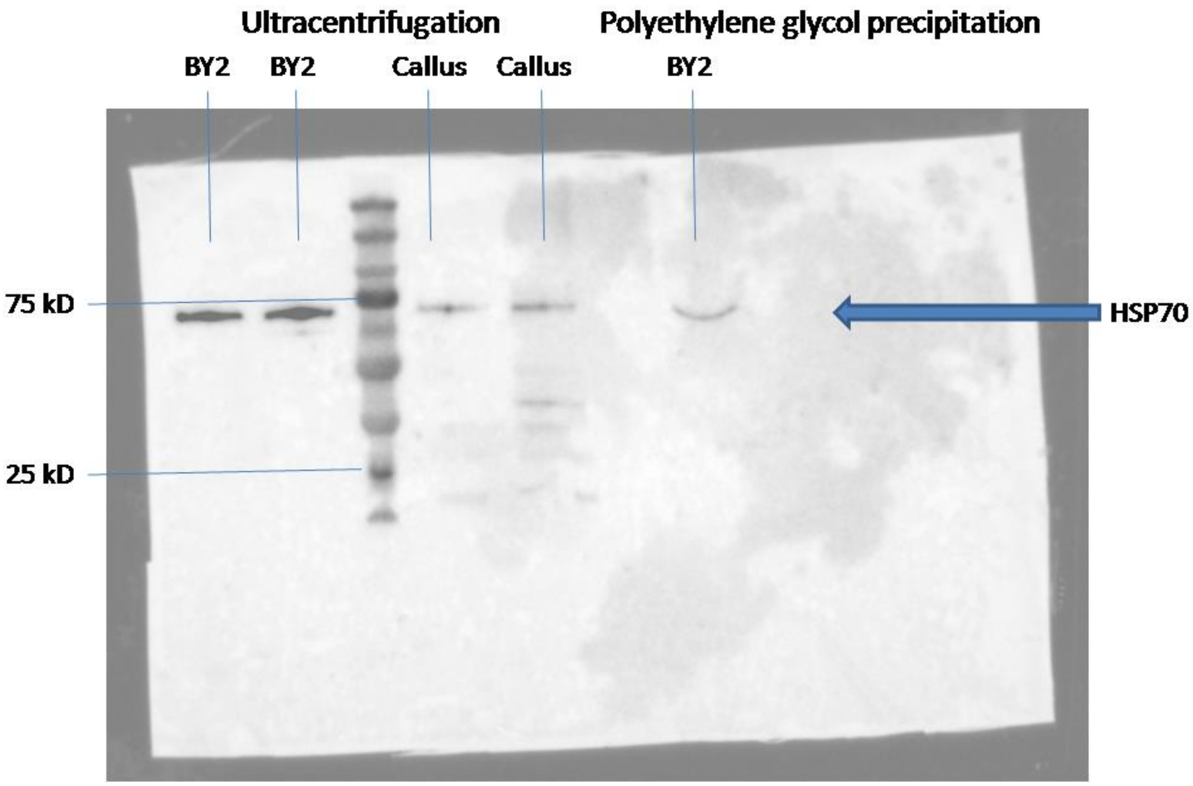

Supplementary Fig. S3: *Nitrocellulose membrane claiming the presence of exosomal protein marker HSP70 in tobacco-derived vesicles isolated from tobacco callus and BY2 suspension cells by ultracentrifugation, as well as tobacco vesicles isolated from BY2 suspension culture by polyetylene glycol precipitation.*
